# RNAi Transfection Optimized in Primary Naïve B Cells for the Targeted Analysis of Human Plasma Cell Differentiation

**DOI:** 10.3389/fimmu.2019.01652

**Published:** 2019-07-23

**Authors:** Tiffany Shih, Saurav De, Betsy J. Barnes

**Affiliations:** ^1^Center for Autoimmune Musculoskeletal and Hematopoietic Disease, Northwell Health, The Feinstein Institute for Medical Research, Manhasset, NY, United States; ^2^Graduate School of Biomedical Sciences Rutgers, The State University of New Jersey, Newark, NJ, United States; ^3^Departments of Molecular Medicine and Pediatrics, Zucker School of Medicine at Hofstra/Northwell Health, Hempstead, NY, United States

**Keywords:** IRF4, AID, siRNA knockdown, plasmablast, antibody secreting cells, B cell

## Abstract

Upon antigen recognition, naïve B cells undergo rapid proliferation followed by differentiation to specialized antibody secreting cells (ASCs), called plasma cells. Increased circulating plasma cells are reported in patients with B cell-associated malignancies, chronic graft-vs.-host disease, and autoimmune disorders. Our aim was to optimize an RNAi-based method that efficiently and reproducibly knocks-down genes of interest in human primary peripheral B cells for the targeted analysis of ASC differentiation. The unique contributions of transcriptional diversity in species-specific regulatory networks and the mechanisms of gene function need to be approached directly in human B cells with tools to hone our basic inferences from animal models to human biology. To date, methods for gene knockdown in human primary B cells, which tend to be more refractory to transfection than immortalized B cell lines, have been limited by losses in cell viability and ineffective penetrance. Our single-step siRNA nucleofector-based approach for human primary naïve B cells demonstrates reproducible knockdown efficiency (~40–60%). We focused on genes already known to play key roles in murine ASC differentiation, such as interferon regulatory factor 4 (IRF4) and AID. This study reports a validated non-viral method of siRNA delivery into human primary B cells that can be applied to study gene regulatory networks that control human ASC differentiation.

## Introduction

B lymphocytes are critical members of the adaptive immune system as they are uniquely capable of secreting high titers of antigen-neutralizing antibody. B cells and their associated antibody-mediated response to antigen are important in the clearance of viral, bacterial, and fungal pathogens. Recognition of these foreign antigens by B cells triggers rapid proliferation and differentiation to specialized antibody secreting cells (ASCs) known as plasma cells. The process of ASC differentiation is a tightly regulated one that relies on synergistic signaling from multiple pathways (1). A large gene-regulatory network of transcription factors is required for regulating this multi-step process. One key player in the differentiation of naïve B cells to ASCs is the transcription factor interferon regulatory factor 4 (IRF4). Its role in ASC differentiation has been well-characterized in mice (2–4). Expression of IRF4 is high in murine ASCs and is critical for upregulating AID and BLIMP1 expression during ASC differentiation to plasma cells (5).

Very few of these murine-based B cell differentiation studies, however, have been replicated in human primary B cells. This delay in data replication is primarily due to difficulties in achieving gene knockdown in human primary naïve B cells, which tend to be more refractory to transfection than immortalized B cell lines, and have been limited by losses in cell viability and ineffective penetrance. While genetic approaches in mice provide invaluable physiological insights for identifying pathways which drive imbalance of B cell subsets, the exclusive use of inbred mice with limited diversity may mask pathways and gene functions that exist uniquely in humans (6, 7). Thus, methods for manipulating gene expression in human primary B cell subsets is essential for transferring findings in mice to humans. More importantly, an *in vitro* approach is necessary to understand how gene dysregulation may contribute to the development of human disease, including post-transplantation systemic persistence of alloimmune and autoimmune responses in chronic graft-vs.-host disease (8–14), as well as the severe consequences of B cell dysfunction in indolently incurable or aggressively fatal B cell-associated malignancies (15, 16), and autoimmunity (17).

In peripheral blood mononuclear cells (PBMCs) isolated from circulating blood, human naïve B cells constitute 0.7–4.9% of leukocytes (18). The variable frequency among individual donors and the refractory nature of primary naïve B cells to gene modification, by lentiviral vector or RNA transfection, have been limiting factors in the study of human ASC differentiation. Gene silencing by transfecting cells with small interfering RNA (siRNA) leads to the rapid degradation of corresponding mRNA and reduced target protein expression. Nucleofection is an electroporation technique that enables efficient introduction of siRNAs into cells and detectable silencing of target genes. Here, we describe an optimized non-viral method for transient knockdown by siRNA delivery into human primary naïve B cells for the study of key genes regulating ASC differentiation and effector function. We focused on genes already known to play a role in murine ASC differentiation, such as IRF4 and AID. This method has been optimized for efficient knockdown of four genes—*IRF4, IRF5, AID*, and *GAPD*—with minimal effects on cell viability and maximal effects on cell recovery and functional analysis after nucleofection.

## Materials and Methods

### Ethics Statement

This study was carried out in accordance with the Declaration of Helsinki. This study used blood from leukopaks of human healthy donors purchased from the New York Blood Center. These types of de-identified, publicly and commercially available specimens are exempt from ethics approval as they are fully anonymized.

### Human PBMC Isolation and Primary B Cell Purification

Peripheral blood mononuclear cells (PBMC) were isolated by Ficoll [Corning, Manassas, VA, 25-072-CV] density centrifugation from buffy coats purchased from the NY Blood Center (Long Island City, NY). Naïve or total B cell purifications was performed by negative selection with magnetic separation according to manufacturer instructions (Stem Cell Technologies, Vancouver, Canada) using EasySep Human naïve B cell enrichment kit [19254] or naïve B cell isolation kit [17254]. Total B cell experiments were performed with cells purified using EasySep Human total B cell enrichment kit [19054] to achieve a >95% enriched population of naïve B cells (CD19^+^IgD^+^) or total B cells (CD19^+^). Isolated naïve B cells ranged from 5 × 10^6^ to 26 × 10^6^ from individual donor leukopaks containing 4 × 10^8^ to 1 × 10^9^ PBMCs.

### Targeted siRNA Nucleofection

Isolated naïve B cells were centrifuged in antibiotic-free, serum-containing media as recommended by the Amaxa P3 Primary Cell 4D-Nucloefector X Kit L [Lonza, Cologne, Germany, V4XP-3024] at 300 × g for 10 min at room temperature. Cells were resuspended in room temperature Amaxa buffer as suggested by the manufacturer for primary cells. 2–3 × 10^6^ cells/100 μL cuvette was the final concentration of cells used for nucleofection. For optimal results, siRNA was resuspended in 1X siRNA buffer composed of 5X buffer [GE Lifesciences, Lafayette, CO, B-002000-UB-100] diluted in nuclease-free water [Ambion, USA, AM9938] and used for nucleofection the same day. Reconstituted siRNA stored at −80°C for up to 2–4 weeks will generally retain knockdown efficiency, as determined by nucleofection and monitoring knockdown efficiency over time (data not shown). B cells were nucleofected with either mock (no siRNA), 1.5 μM of ON-TARGETplus Non-targeting Control Pool [Dharmacon, Lafayette, CO, D-001810-10-05] or SMARTpool ON-TARGETplus human *IRF4* siRNA [Dharmacon, LU-019668-00-0005]. 1–1.5 μM ON-TARGETplus Targeted Control *GAPD* Pool [Dharmacon, D-001830-10-05], 1–1.5 μM of ON-TARGETplus *AICDA* siRNA [Dharmacon, LU-021409-00-0005], and 1.5 μM siGLO green transfection indicator siRNA [Dharmacon, D-001630-01-05] were also used. Cells were nucleofected using program EO-117 for primary human B cells of the Amaxa 4D Nucleofector system [Lonza] composed of the core unit and the X unit.

Immediately after nucleofection, 500 μL of pre-warmed (37°C) antibiotic-free media (10% fetal bovine serum (FBS) in Iscove's Modified Dulbecco's Media (IMDM) without antibiotics) was added to the cuvette by slowly releasing the media along the wall of the cuvette. The final suspension was then transferred into wells of a 24-well plate that each contained 1 mL of pre-warmed antibiotic-free media [Sigma, USA, F4135] per cuvette and cells allowed to rest in culture for 24 h at 37°C in 5% CO_2_. After resting, cells were transferred to a 14 mL Falcon tube to be pelleted, counted and then cultured with the appropriate cocktails for B cell activation or plasmablast differentiation.

### Viability Post-nucleofection

Viability was determined by staining cells with trypan blue [Life Technologies, Carlsbad, CA, 15250-061] after resting nucleofected cells for 24 h and assessing by hemocytometer. Percent viable was calculated using the equation 100 × (total cells—blue cells)/total number.

### *In vitro* B Cell Activation and Plasmablast Differentiation

After resting, nucleofected naïve B cells were cultured in 96-well U-bottom plates [Costar, USA, 3799] at a minimal density of 0.5 × 10^6^ in 250 μL of IMDM supplemented with 10% FBS and 1X penicillin-streptomycin [Corning, 30-002-Cl] per well. B cell cultures of 3 days or less were treated with or without 10 μg/mL unconjugated goat anti-human IgM antibody [Southern Biotech, Birmingham, AL 2020-01] and 2.5 μg/mL CpG-B oligodeoxynucleotide (ODN) 2006 [Hycult Tech, Uden, The Netherlands, HC4309]. For plasmablast differentiation, purified naïve B cells were cultured for 7 days in the presence of 200 ng/mL sCD40L [Peprotech, Rocky Hill, NJ 310-02-10UG] alone or a “C4” cocktail, consisting of 200 ng/mL sCD40L, 100 ng/mL IL-21 [Peprotech, 200-21-2UG], 10 μg/mL unconjugated goat anti-human IgM antibody, and 2.5 μg/mL CpG-B ODN 2006 (17, 19–24). For activation prior to nucleofection, 2 × 10^6^ cells/mL were stimulated in a 24-well flat bottom plate overnight with or without CpG-B plus anti-IgM in a final volume of 1 mL IMDM supplemented with 10% FBS and penicillin-streptomycin. After pre-activation, cells were washed twice with 0.5% BSA in 1 × PBS and counted for the nucleofection protocol described above.

### Flow Cytometry

B cells were washed with 1X PBS and stained with Live/Dead Fixable Yellow Dead Cell Stain Kit viability discrimination dye [Life Technologies, L34959]. Cells were subsequently blocked in 2% bovine serum albumin (BSA) supplemented with human TruStain FcX Blocker [Biolegend, San Diego, CA, 422302] for 5 min and then stained with antibodies against B cell surface markers (Table 1). After staining, cells were washed in 1X PBS and then fixed in 2% PFA before analysis on a BD Fortessa flow cytometer [BD Biosciences]. For intracellular protein staining, after overnight fixation, cells were permeabilized in 0.1% Triton X-100 and rinsed in 1X PBS 2 times before blocking in 5% BSA solution. Intracellular AID was detected with unconjugated goat polyclonal primary antibody [Santa Cruz Biotech., CA, sc-14680], and subsequently stained with donkey anti-goat IgG-AF488 secondary [Invitrogen, USA, A-11055]. Intracellular GAPD was stained with unconjugated rabbit anti-human GAPDH antibody EPR16891 [Abcam, USA, ab181602] and subsequently with goat anti-rabbit AF488 [Life Technologies, A11034]. Intracellular IRF4 was detected with rat anti-human/mouse IRF4-phycoerythrin (PE) [Biolegend, 646404] and rat immunoglobulin (Ig)G1, k isotype control [Biolegend, 400408]. Cells staining positive for the live/dead stain were excluded from the flow cytometry analysis. Doublets were excluded from our analysis of FSC-A vs. FSC-H gating. Naïve B cells were defined by CD19^+^IgD^+^ surface expression, and plasmablasts were defined by CD19^+^CD20^+^IgD^−^CD27^+^CD38^+^ surface expression (Supplementary Figure 1). FCS files were analyzed using FlowJo v9.3.2 (Tree Star Inc., Ashland, OR).

**Table 1:** Antibodies used for surface staining and flow cytometry.

**Surface marker**	**Fluorochrome**	**Clone**	**Company**	**Catalog**
Live/dead	yellow	N/A	Invitrogen/thermofisher	L34968
Live/dead	green	N/A	Invitrogen/thermofisher	L34969
CD19	BV421	HIB19	Biolegend	302234
CD19	BV510	HIB19	Biolegend	302242
IgD	APC	IA6-2	Biolegend	348222
CD38	PE-CF594	HIT2	BD biosciences	562288
CD27	PE	M-T271	BD pharmigen	555441

### Statistical Analysis

All statistical analyses were performed using Prism v6.2 (GraphPad Software, San Diego, CA). Student's *t*-test was used for comparisons between two samples with normal distribution. Prior to test, graph kurtosis was analyzed to ensure normal distribution. Data are reported as mean ± SEM. *P*-value < 0.05 was considered significant.

## Results

### Analysis of Cell Viability After Nucleofection and Optimization of Cell Number for ASC Differentiation

**Figure 1:**
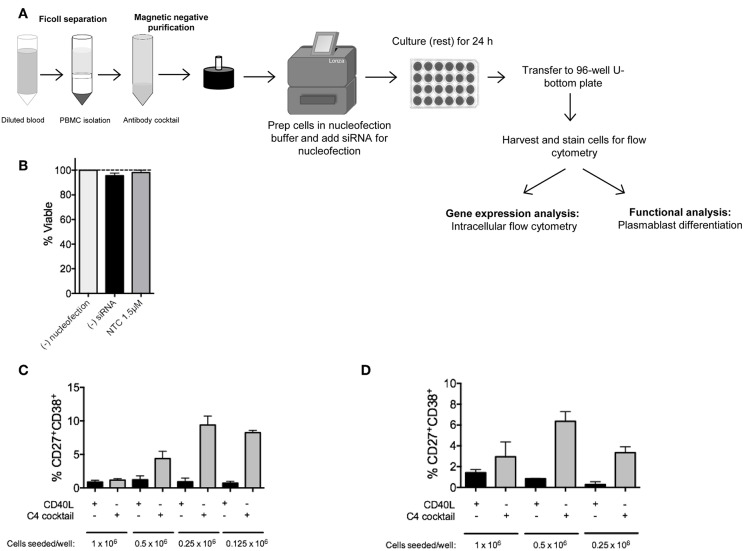
RNAi knockdown in primary human naïve B cells by Amaxa nucleofection protocol. **(A)** Overview of nucleofection protocol and experimental readouts. **(B)** Isolated human naïve B cells were nucleofected with or without non-targeting control (NTC) siRNA and rested for 24 h. Viability of cells after resting was determined by trypan blue staining (*n* = 4). **(C)** Optimal seeding density of non-nucleofected primary human naïve B cells to generate plasmablasts in a 96-well plate via 7 day *in vitro* culture with C4 cocktail (*n* = 4). **(D)** Same as **(C)** except optimal seeding density is shown for nucleofected cells (*n* = 4).

We previously attempted shRNA lentiviral transduction of human primary naïve B cells and were unsuccessful. We later developed a siRNA nucleofection protocol that required two rounds of nucleofection with low concentrations of *IRF5* siRNAs over 48 h to obtain ~40–60% knockdown efficiency of IRF5 proteins in human primary naïve B cells (17). We have now optimized the protocol further for single siRNA nucleofection and knockdown of other genes involved in ASC differentiation (Figure 1A). We initially optimized the protocol for IRF4 knockdown, as it is known to play essential roles in murine ASC differentiation. In mice lacking *Irf4*, B and T cells were unable to proliferate in response to B cell receptor (BCR), T cell receptor (TCR), CD40, or LPS stimulation (25). Studies in mice revealed that *Irf4* is necessary for AID upregulation, class-switch recombination (CSR), and generation of plasma cells in response to BCR signaling (5, 26, 27).

Since primary B cells are notoriously difficult-to-transfect, and we are interested in one of the more rare subsets, plasma cells, we first optimized naïve B cell numbers and viability after nucleofection for downstream analysis of plasma cells by flow cytometry (17). In this assay, cells were either left untouched, mock-nucleofected, or nucleofected with 1.5 μM ON-TARGETplus Non-targeting Control (NTC) Pool siRNA. By trypan blue staining, no significant difference in cell viability was detected 24 h post-nucleofection of primary naïve B cells (Figure 1B).

A single leukopak of blood (~35–38 mL) yields between 5 and 26 × 10^6^ naïve B cells. To determine the appropriate number of purified primary naïve B cells that will lead to sufficient plasma cell numbers for functional analysis, we examined differentiation of naïve B cells, at different seeding densities, to plasmablasts by 7 day *in vitro* culture with C4 cocktail (anti-IgM, CpG-B, IL21, and CD40L) (17, 19–24, 28). Similar to previously published work, in non-nucleofected naïve B cells, a seeding density of 0.25 × 10^6^ cells per well of a 96-well plate was required for the optimal generation of CD19^+^IgD^−^CD27^+^CD38^+^ plasmablasts (Figure 1C) (29, 30). Representative gating strategy for plasmablasts is shown in Supplementary Figure 1. As recommended by the manufacturer, all Amaxa nucleofection reagents were kept at room temperature prior to use. For primary naïve B cells, we found that an optimal cell concentration for nucleofection with maximal siRNA entry and cell viability post-nucleofection was 2–2.5 × 10^6^ cells per cuvette (in 100 μL volume); below 1.5 × 10^6^ or above 3 × 10^6^ million cells reduced viability and nucleofection efficiency. After nucleofection, 500 μL of 37°C pre-warmed IMDM supplemented with 10% FBS and no antibiotics was added to each cuvette and gently aspirated with pipettes provided in the kit for gentle transfer of cells into wells of a 24-well plate that had been pre-equilibrated to 37°C at 5% CO_2_ with 1.0 mL of IMDM/well. Cells were then rested for 24 h at 37°C in 5% CO_2_. Cell loss can occur at this point when transferring from the 24-well plate to the tube for washing. To address this, wells were thoroughly washed with 1 mL sterile 1X PBS or media using a 1,000 μL pipette to physically detach cells along the bottom surface and the circumference of the well. This step can be repeated as needed. Distinct from non-nucleofected naïve B cells, we found that 0.5 × 10^6^ cells per well of a 96-well plate were required for optimal plasmablast generation after mock nucleofection (Figure 1D) (17). Thus, a critical step in the process is to re-count your cells after nucleofection and 24 h resting before transferring naïve B cells to a 96-well plate for 7 day *in vitro* culture to plasmablasts using the C4 cocktail. A good rule of thumb for calculating cell number for downstream functional analysis is to begin with nearly twice the number of cells that you want to end with for functional analysis post-nucleofection.

### Analysis of Knockdown Efficiency and ASC Differentiation

IRF4 expression is high in plasma cells and low in naïve B cells but expression increases within 48 h after stimulation with anti-IgM for BCR cross-linking or CD40L (31, 32). siRNA-mediated knockdown of *IRF4* has been previously described in total CD19^+^ B cells using a final concentration of 1.5 μM siRNA (33). We thus used *IRF4* siRNAs in the range of 1–1.5 μM for knockdown in human primary naïve B cells. Unlike IRF5 that is expressed at sufficient basal levels in naïve B cells to detect knockdown without stimulation (17), both IRF4 and AID are expressed at very low levels and thus require B cell activation to detect knockdown. We found that IRF4 expression peaked at 48 h in CD19^+^IgD^+^ B cells after stimulation of PBMC with anti-IgM and the TLR9 agonist CpG-B; AID expression peaked at 72–96 h (Supplementary Figures 2A,B). We thus used these time points for analysis of IRF4 and AID protein expression after knockdown. An *a priori* understanding of mRNA and protein expression patterns of the particular gene of interest is required to determine appropriate time points for knockdown analysis.

**Figure 2:**
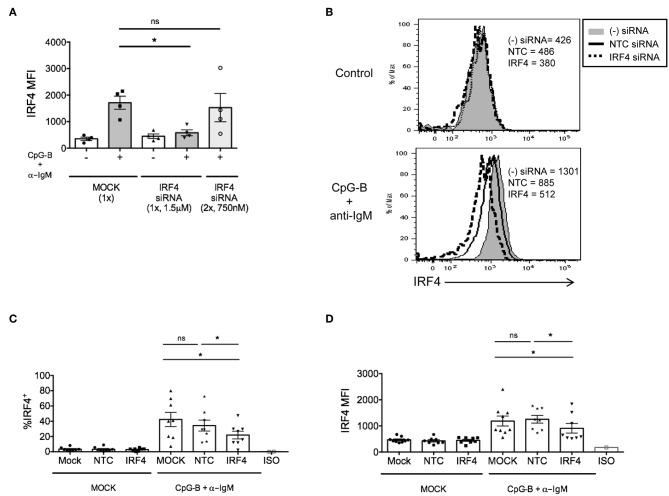
IRF4 knockdown in primary human naïve B cells by Amaxa nucleofection protocol. Isolated human naive B cells were nucleofected with or without *IRF4* targeting siRNA and stimulated with or without anti-IgM + CpG-B for 48 h. **(A)** Comparison of single vs. dual nucleofection protocols (*n* = 4). (B) Isolated human naive B cells were nucleofected with or without 1.5 μM *IRF4* targeting siRNA or non-targeting control (NTC) siRNA, rested for 24 h, and stimulated with or without anti-IgM + CpG-B for 48 h. Representative histogram overlays show IRF4 expression after stimulation. **(C,D)** Similar to **B** except data are summarized from multiple independent experiments showing % IRF4^+^ CD19^+^IgD^+^ B cells **(C)** and MFI of IRF4 in CD19^+^IgD^+^B cells **(D)** from *n* = 8 independent donors. Paired *t*-test for significance was performed (*p < 0.05).

Although we previously found that efficient knockdown of IRF5 in human primary naïve B cells required a dual nucleofection protocol with low siRNA concentrations (17), for IRF4, we were able to optimize knockdown by single nucleofection of 1.5 μM siRNAs (Figure 2A). Using the single nucleofection protocol, we examined IRF4 knockdown efficiency after single nucleofection with mock, SMARTpool ON-TARGETplus human *IRF4* siRNA or NTC siRNA. Results revealed a range of 30–50% knockdown of IRF4 proteins by *IRF4* siRNA, and not NTC siRNA, at 72 h post-nucleofection (24 h rest plus 48 h stimulation) (Figures 2B–D and Supplementary Figures 2C–F) with >95% post-nucleofection viability (Supplementary Figure 3A). Given that IRF4 knockdown with *IRF4* siRNAs gave a Gaussian distribution of knockdown levels (Figure 2B), these data suggest that all cells, rather than a small subset of cells, were nucleofected with siRNA.

**Figure 3:**
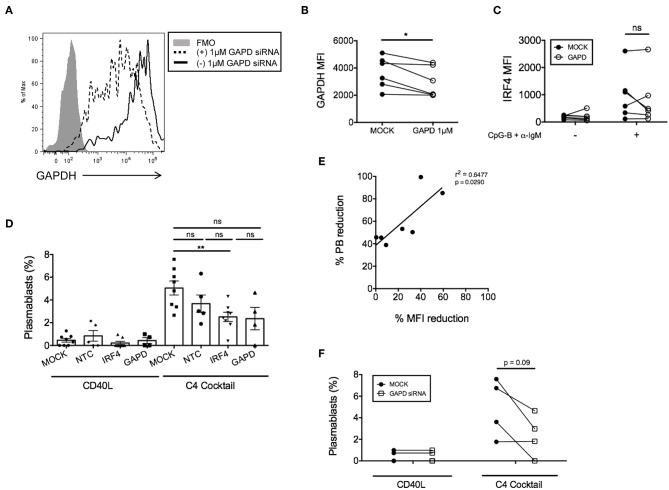
GAPD housekeeping gene as a positive control for nucleofection. **(A)** Representative figure showing histogram overlay of B cells nucleofected with 1 μM *GAPD* siRNA and then cultured for 48 h post-nucleofection. **(B)** Paired dots show the efficiency of GAPDH knockdown in CD19^+^IgD^−^ B cells from matched independent donors after 48 h culture (*n* = 6). **(C)** Same as **(B)** except IRF4 expression was determined after nucleofection with 1 μM *GAPD* siRNA (*n* = 6). **(D)** Naïve B cells were cultured for 7 days with the C4 cocktail (anti-IgM + CpG-B + IL-21+ CD40L) to induce plasmablast differentiation post-nucleofection with 1.5 μM *GAPD* siRNA, 1.5 μM *IRF4* siRNA or 1.5 μM NTC siRNA. Plasmablast differentiation was determined by flow cytometry analysis of CD19^+^IgD^−^CD27^+^CD38^+^ cells (*n* = 9). Bars represent mean ± SEM. **(E)** Correlation between % plasmablast reduction and % IRF4 MFI reduction. **(F)** Effect of *GAPD* siRNA on % plasmablasts from **(D)** are shown as paired dots to indicate matched donor effects. Paired *t*-test for significance was performed (*p < 0.05).

In addition to negative control siRNAs, positive control siRNAs are recommended to confirm knockdown efficiency and specificity of siRNA function (34). We utilized a positive control siRNA targeting *GAPD* (ON-TARGETplus *GAPD* Control Pool siRNA). All targeted and non-targeted siRNAs were used at the same concentration for direct comparison of effects (either 1 or 1.5 μM) (Supplementary Figure 3B). Nucleofection with *GAPD* siRNAs resulted in a significant reduction in GAPD protein at 72 h post-nucleofection (24 h rest plus 48 h stimulation) (Figures 3A,B), with little significant impact on IRF4 expression (Figure 3C) or post-nucleofection viability (Supplementary Figure 3A). Notably, GAPD siRNA also provided a Gaussian distribution of knockdown levels, suggesting that all cells are getting nucleofected with siRNAs equally. ASC differentiation was then examined after mock-nucleofection, nucleofection with *IRF4* siRNA, NTC siRNA, or *GAPD* Targeted siRNA. Similar to findings in *Irf4*^−/−^ mice, knockdown of IRF4 in human primary naïve B cells correlated with a significant reduction (~30–40%) in ASC differentiation (Figures 3D,E). While NTC siRNA had no significant effect on plasmablast generation, we were surprised to detect a strong trend in plasmablast reduction after GAPD knockdown suggesting a potential role for GAPD in plasma cell differentiation (Figures 3D,F). Indeed, GAPD is an important regulator of cell growth, proliferation and survival due, in part, to its role in regulating the generation of glycolytic ATP (35–38). A subpopulation of naïve B cells, in response to B cell activation, will survive and undergo clonal expansion, CSR and differentiation to ASCs, in which GAPD has been implicated in Migliaccio et al. (39). Thus, while positive control siRNAs can serve as important tools for determining specificity of function, they can also complicate the system, depending on the downstream assays used to determine functional consequence(s) of siRNA knockdown. Nonetheless, data clearly show that the single nucleofection protocol can be used to knockdown IRF4 efficiently, leading to a significant reduction in human ASC differentiation.

### Applying the Single Nucleofection Knockdown Protocol to Other Genes Involved in ASC Differentiation

**Figure 4:**
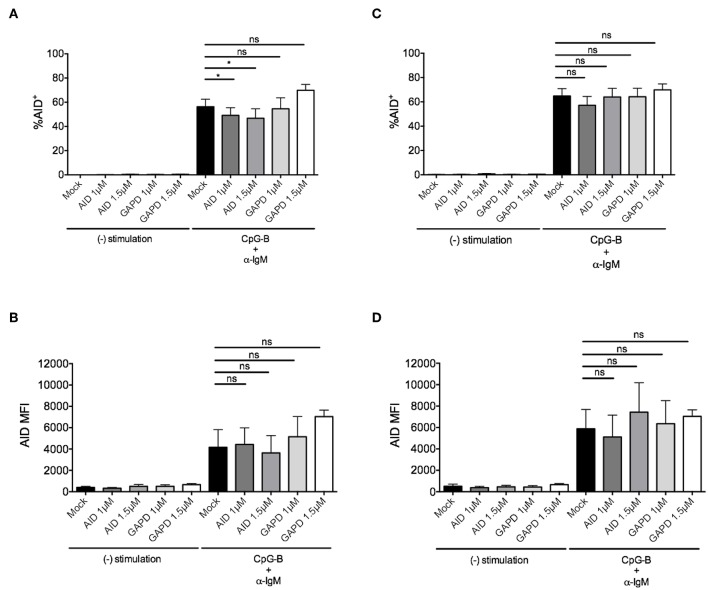
Efficiency of AID knockdown in primary naïve human B cells is marginal at 48 and 72 h post-nucleofection. Isolated naïve B cells were stimulated with anti-IgM + CpG-B after single nucleofection protocol with *AID* or *GAPD* siRNA (1 or 1.5 μM). **(A)** % AID^+^CD19^+^IgD^+^ B cells is shown at 48 h post-stimulation. **(B)** AID MFI is shown at same time point as **(A)**. **(C)** Same as **(A)** except % AID^+^CD19^+^IgD^+^ B cells is shown 72 h post-stimulation. **(D)** AID MFI is shown at same time point as **(C)**. Bars represent mean ± SEM (*n* = 4). Paired *t*-test for significance was performed (*p < 0.05).

To determine whether this method is effective for silencing other genes that are induced after B cell activation, we examined knockdown of AID, a factor that is critical for CSR during ASC differentiation (40, 41). After titration, we identified the optimal concentration of 1–1.5μM *AICDA* siRNA that provided a significant, albeit, small reduction in the percentage of CD19^+^IgD^+^AID^+^ B cells (~10–20%) after anti-IgM + CpG-B stimulation for 48 h (Figure 4A). Somewhat surprising, this reduction did not translate into a significant reduction in AID MFI (Figure 4B). Based on AID expression kinetics (Supplementary Figure 2B), we extended the activation time point to 72 h and were still unable to detect significant effects on AID expression (Figures 4C,D).

**Figure 5:**
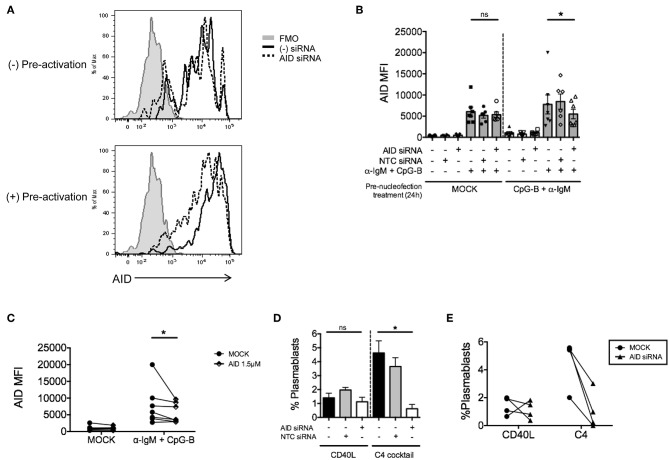
Pre-activation of primary naïve human B cells enhances the effect of AID knockdown. Isolated naïve B cells were cultured for 24 h (+Pre-activation) or without (-Pre-activation) anti-IgM + CpG-B then nucleofected one time with *AID* or NTC siRNA (1.5 μM). After 24 h resting, cells were re-stimulated with or without anti-IgM + CpG-B for 72 h. **(A)** Representative histogram overlay of AID expression after knockdown in the presence or absence of pre-activation. **(B)** AID MFI in CD19^+^IgD^+^ B cells was determined at 72 h post-stimulation (*n* = 7). **(C)** Paired dots show the efficiency of AID knockdown in CD19^+^IgD^−^ B cells from matched independent donors in **(B)**. **(D)** Following pre-activation and nucleofection with *AID* or NTC siRNA, cells were *in vitro* cultured for 7 days with C4 cocktail to induce plasmablast differentiation (CD19^+^IgD^−^CD27^+^CD38^+^) (*n* = 4). **(E)** Effect of *AID* siRNA on % plasmablasts from **(D)** are shown as paired dots to indicate matched donor effects. Bars represent mean ± SEM. *P*-values were determined by paired *t*-test for significance (*p < 0.05).

Due to the low level of AID knockdown observed, we examined an alternate approach to achieve more robust knockdown of AID expression for functional analysis. We hypothesized that the inherent characteristics of the gene might require activation prior to nucleofection. Thus, purified naïve B cells were activated first with anti-IgM and CpG-B overnight prior to the standard nucleofection protocol and re-stimulation. Indeed, we detected a stronger knockdown effect after pre-activation (Figure 5A), revealing that 1.5 μM *AICDA* siRNA provides a significant reduction in AID MFI (ranging from ~30–50% knockdown) (Figures 5B,C). This level of AID knockdown resulted in ~90% loss of plasmablast differentiation (Figures 5D,E), with no significant effect on cell viability (Supplementary Figure 3A). Altogether, these data indicate that the single nucleofection protocol for knockdown in human primary naïve B cells can be applied to multiple genes.

### Sorting of Knockdown Cells by Co-nucleofection With siGLO

It was previously reported that the abundance of target gene expression is a critical factor that determines the efficiency of siRNA-mediated gene silencing (42). *IRF4* and *GAPD* siRNAs showed a Gaussian distribution of knockdown levels suggesting that most cells were nucleofected equally with siRNAs (Figures 2B, 3A), while AID siRNAs showed a more disparate level of knockdown distribution. Given that gene expression can vary based on cellular context resulting in unequal knockdown effects, we attempted to optimize a method of co-nucleofection for sorting and functional analysis of nucleofected cells with knockdown.

**Figure 6:**
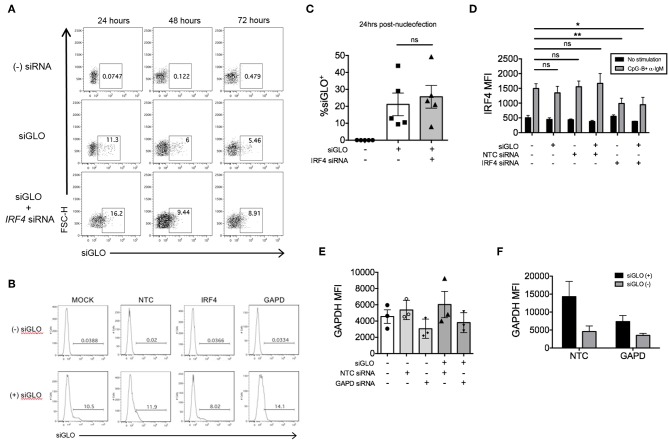
siGLO green nucleofection of purified primary naïve B is transient and lost by 48 h post-nucleofection. **(A)** Representative dot plots showing purified naïve B cells nucleofected with 1.5 μM siGLO or 1.5 μM siGLO + 1.5 μM *IRF4* siRNA cultured for 24, 48, and 72 h after nucleofection and analyzed for siGLO^+^ cells. **(B)** Similar to **(A)** except representative histograms show the different siGLO co-nucleofection efficiencies in naïve B cells at 24 h post-nucleofection. **(C)** % siGLO^+^ B cells are shown from multiple independent experiments of **(B)** at 24 h post-nucleofection (*n* = 5). **(D)** IRF4 knockdown effect is retained in naïve B cells co-nucleofected with siGLO and *IRF4* siRNA after stimulation with anti-IgM + CpG-B for 48 h (*n* = 4). **(E)** Same as **(D)** except GAPD MFI was examined 24 h post-nucleofection (*n* = 3). **(F)** Comparison of GAPD knockdown from **(E)** in siGLO^+^ vs. siGLO^−^ cells. Bars represent mean ± SEM. *P*-values were determined by paired *t*-test for significance (*p < 0.05, **p < 0.01).

We previously explored co-nucleofection of *IRF5* siRNA with GFP mRNA (Trinity Biotech: L601) or pmaxGFP^TM^ vector (Lonza) as a method to sort for cells with knockdown (17). Unfortunately, we were unsuccessful as co-nucleofection with GFP mRNA resulted in ~20% GFP^+^ naïve B cells with no correlation between GFP and IRF5 knockdown; both GFP^+^ and GFP^−^ cells showed equivalent IRF5 knockdown levels (17). Similarly, the pmaxGFP^TM^ resulted in a low yield of GFP^+^ cells with only ~2–4% of naïve B cells expressing GFP (17). At the time, we hypothesized that the failed attempts might be due to size restrictions on B cell uptake; green fluorescent protein (GFP) is larger than most siRNAs. Here, we attempted a new strategy for knockdown and selection using Dharmacon's siGLO green reagent that is used to examine siRNA transfection efficiency. Primary naïve B cells were co-nucleofected with equal parts of *IRF4* siRNA and siGLO green. After 24 and 48 h post-nucleofection, nucleofecion efficiency and IRF4 knockdown were examined by flow cytometry. We detected a range in siGLO nucleofection efficiency with 10 to 45% of cells being siGLO^+^; similar levels were seen in co-nucleofected cells (Figures 6A–C). Importantly, IRF4 knockdown levels were similar between co-nucleofected and *IRF4* siRNA nucleofected samples (Figure 6D) suggesting that siGLO does not compete with IRF4 for entry into cells during nucleofection. Unfortunately though, we found that the siGLO signal dramatically decreases, independent of concentration, 24 h post-nucleofection (Figure 6A), which is before we are able to detect significant IRF4 knockdown (Figure 2). Thus, to further assess the use of siGLO for tracking nucleofection with knockdown, we co-nucleofected cells with siGLO and NTC siRNA or *GAPD* siRNA and performed the similar analysis at the earlier time point of 24 h post-nucleofection. We detected similar siGLO nucleofection efficiency as seen before (~10–30%, Supplementary Figure 4A) and knockdown of GAPD was retained independent of siGLO (Figure 6E). Importantly, while the overall nucleofection efficiency was low with siGLO (Supplementary Figure 4B), data in Figure 6F suggest that knockdown of genes with abundant baseline expression (Supplementary Figure 4C) may be tracked with siGLO since we detected ~40% reduction of GAPD protein levels in siGLO^+^ cells at 24 h post-nucleofection.

**Figure 7:**
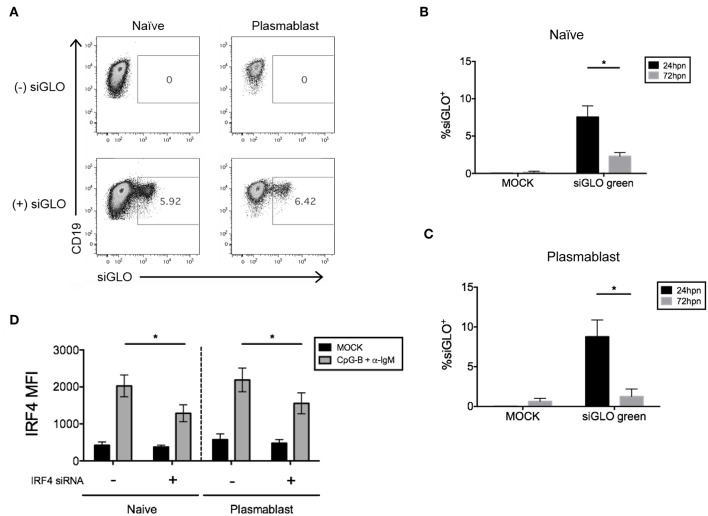
Efficiency of siGLO co-nucleofection with *IRF4* siRNA in purified total CD19^+^ human B cells. **(A)** Representative dot plots of gated naïve B cells (CD19^+^IgD^+^) and plasmablasts (CD19^+^IgD^−^CD27^+^CD38^+^) that are siGLO^+^ at 24 h post-nucleofection with 1.5 μM siGLO. **(B,C)** Similar to **(A)** except data are summarized from multiple independent experiments showing siGLO nucleofection efficiency at 24 and 72 h post-nucleofection in naïve B cells **(B)** and plasmablasts **(C)** (*n* = 4). **(D)** IRF4 knockdown efficiency is retained in naïve B cells and plasmablasts after co-nucleofection of total B cells with siGLO and stimulation with anti-IgM + CpG-B for 48 h (*n* = 4). Bars represent mean ± SEM. *P*-values were determined by paired *t*-test for significance (*p < 0.05).

We next attempted co-nucleofection of total CD19^+^ B cells with siGLO and *IRF4* siRNA to examine nucleofection efficiency and knockdown in multiple B cell subsets at one time. We detected similar but low levels of siGLO in both naïve B cells and plasmablasts 24 h post-nucleofection (Figures 7A–C). Analysis of IRF4 knockdown in naïve B cells revealed a similar knockdown level as that seen by nucleofection of purified naïve B cells (Figures 2, 7D) suggesting that this may be an alternate method for knockdown in naïve B cells. However, depending on the experimental outcome, sorting naïve B cells from nucleofected total B cells is unlikely to yield sufficient cell number for downstream functional analysis. Last, significant knockdown of IRF4 in plasmablasts was detected also by this method.

## Discussion

In this report, we describe an optimized method for RNAi nucleofection of human primary naïve B cells to study the role of genes, such as IRF4 and AID that contribute to human ASC differentiation. The knockdown efficiency of both IRF4 and AID was sufficient to observe downstream functional effects on plasmablast differentiation. The central issue in optimizing gene knockdown, however, is to understand basal expression and expression induced after stimulation of your target gene. For transcription factors such as IRF5 that are sensitive to activation by nucleic acid-sensing innate immune sensors (43–47), we suggest optimizing with the low dose dual nucleofection protocol described by De et al. (17). Low concentrations of siRNA (500 nM), in two sequential nucleofections, provided an IRF5 knockdown with 40–60% efficiency (17). The lower siRNA concentrations likely minimize activation of RNA sensors and genes regulating the inflammatory response, which ultimately lead to IRF5 upregulation (48–50). In the case of IRF4, we were unable to detect knockdown after dual nucleofection with low concentrations of siRNA (Figure 2A). Dual nucleofection also results in more cell loss due to two transfer steps from cuvette to plate (data not shown). Further, genes such as IRF4 and AID that are expressed at low levels in naïve B cells require stimulation with a B cell activating trigger in order to detect knockdown. While optimizing our protocol for AID, we found that pre-activation was necessary to enhance the effect of knockdown. Similarly, other examples exist for genes with distinct patterns of expression in B cell differentiation, such as *BACH2* (51), that requires alternate nucleofection protocols. Thus, the variation in knockdown efficiency between genes and amongst methods to determine knockdown efficiency (MFI vs. percent positivity) may be attributed to the inherent variation in gene expression (42).

**Figure 8:**
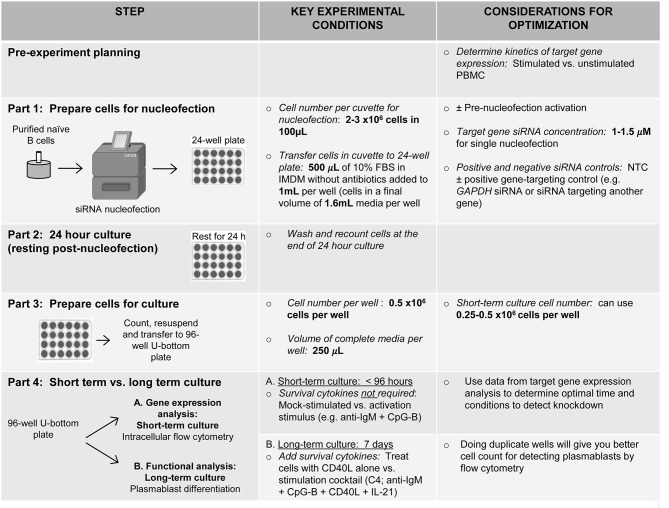
Schematic summarizing an optimized method for RNAi knockdown in primary human naïve B cells.

Despite the limited and variable quantity of starting material, combined with the limited recovery of cells post-nucleofection, the bulk study of transient gene knockdown in human primary naïve B cells by flow cytometry after nucleofection can be achieved with the methods described herein (Figure 8). The optimal cell density of nucleofected naïve B cells when cultured long-term for plasmablast differentiation (7 days) was 5 × 10^5^ after nucleofection. This number is likely necessary due to cell-cell contact required by B cells. Thus, key features of our method take into account cell density at the point of nucleofection and after nucleofection, as well as the kinetics of the target protein expression.

A good starting point for knocking down any gene of interest is to begin with 1 μM of siRNA; however, this may require further optimization depending on the kinetics of gene expression. Data presented herein suggests that knockdown in other B cell subsets can be obtained by nucleofection of total B cells, followed by sorting subsets of interest (Figure 7). However, some subsets, such as memory B cells and plasma cells that are low in the circulation will require alternative methods (52) for knockdown. For human primary GC plasma cells, Maarof et al. (53) describe a method for isolation and nucleofection to study IL-24 cytokine expression. Additional methods have been developed to study pre-B cells (54). Whether the general approach we describe can be applied to studies in patient samples is dependent on the specific considerations regarding amount of starting material available and relative frequency of the cell type being studied (55–58). Thus, depending on the cell subset of interest and functional read-out, methods may need to be further optimized to take into account reduced cell numbers.

Unfortunately, 100% transfection efficiency was not achieved in our study or by others (17, 51–54), possibly due to stochastic kinetics of siRNA entry into primary B cells (59). As death of primary human B cells following nucleofection correlates with size and structure of nucleic acids being transfected (60), siRNA knockdown efficiencies may vary between siRNAs and gene targets (61). Further, nucleofection of human B cells has been reported to be relatively inefficient when compared to human T cells (52). And, few, if any, studies have reported siRNA knockdown in human primary naïve B cells (17, 51). Applications of new technologies for genome editing, such as CRISPR-CAS9, in primary total B cells have reported comparable on-target efficacies as siRNA knockdown (62–64). We thus propose that the variables described in our optimized method using RNAi transfection technologies will provide a complementary approach for autologous therapeutic genome editing where stable modification to the host genome may entail long-term safety risks to the recipient. Further, these methods can be used more rapidly than CRISPR-CAS9 technology to strengthen the translation of findings from non-human models to human disease models.

## Data Availability

All relevant data generated and analyzed for this study are included in the manuscript and Supplementary Files. For additional protocol details and/or original data, please contact Betsy J. Barnes (bbarnes1@northwell.edu).

## Ethics Statement

This study was carried out in accordance with the Institutional Review Board of Rutgers Biomedical and Health Sciences and the Feinstein Institute for Medical Research with written consent from all subjects. All subjects gave written informed consent in accordance with the Declaration of Helsinki.

## Author Contributions

SD, TS, and BB were involved in the conception and design of the methodology and wrote the manuscript. TS performed all experiments. TS and BB analyzed the data. All authors have read and approved the final manuscript.

### Conflict of Interest Statement

The authors declare that the research was conducted in the absence of any commercial or financial relationships that could be construed as a potential conflict of interest.
