# Supplementary material for: RNAi Transfection Optimized in Primary Naïve B Cells for the Targeted Analysis of Human Plasma Cell Differentiation
Source: Front Immunol. 2019 Jul 23;10:1652. doi: 10.3389/fimmu.2019.01652 (PMC6664017; doi:10.3389/fimmu.2019.01652)
Supplement: Supplementary file 1 [file Data_Sheet_1.pdf]

Supplemental Figures and Legends

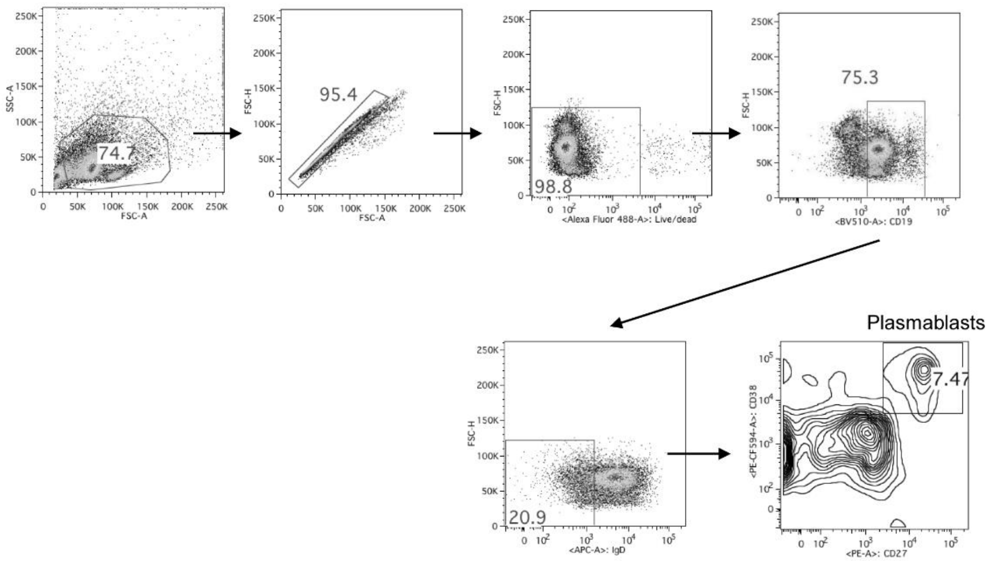

SF1. Representative gating strategy for plasmablasts after 7 day *in vitro* culture with the C4 cocktail.

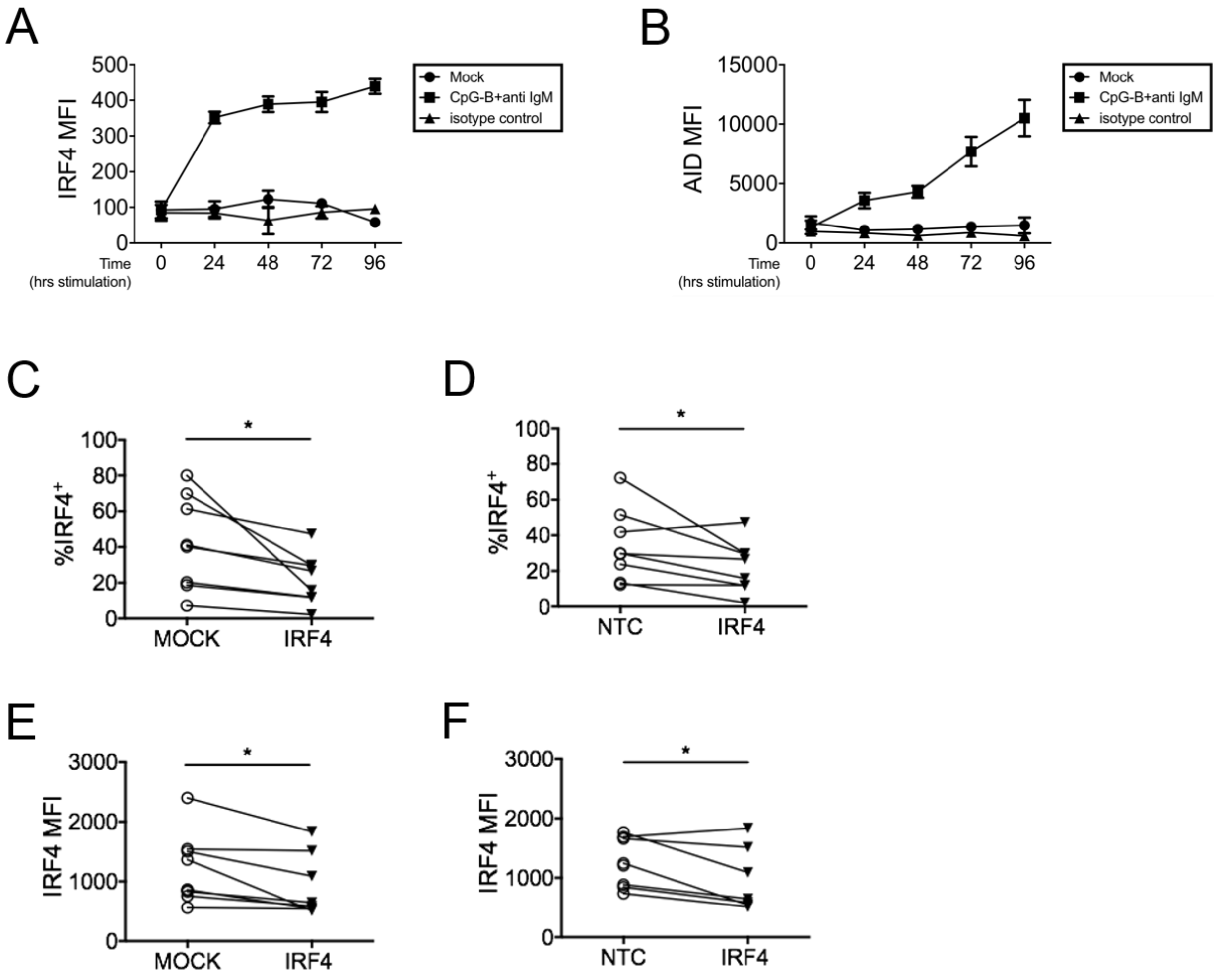

SF2. Kinetics of IRF4 and AID expression in CD19+IgD+ B cells and IRF4 knockdown. IRF4 (**A**) and AID (**B**) MFI in CD19$^+$IgD$^+$ B cells after stimulation of 2x10^6 cells/mL with or without CpG-B and anti-IgM for 0, 24, 48, 72 and 96 hours (n= 4). **C,D**. Data are from **Fig. 2C**. Isolated human naïve B cells were nucleofected with or without 1.5µM *IRF4* targeting siRNA or non-targeting control (NTC) siRNA, rested for 24 hours, and stimulated with or without CpG-B + anti-IgM for 48 hours. % IRF4$^+$CD19$^+$IgD$^+$ B cells in mock-nucleofected (**C**) or NTC-nucleofected (**D**) versus *IRF4* siRNA-nucleofected cells is shown as paired dots to represent matched independent donors; n=8. **E,F**. Same as **C,D** except data are from **Fig. 2D** showing IRF4 MFI as paired dots for matched donors; n=8. Paired t-test for significance was performed (*p<0.05).

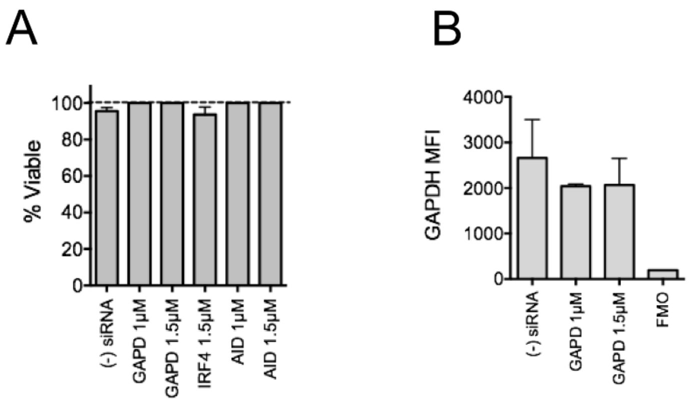

SF3. Cell viability after knockdown and GAPD knockdown efficiency. Isolated human naïve B cells were nucleofected with or without the indicated smart pool siRNAs and rested for 24 hours after nucleofection. **A.** Viability of cells was determined by trypan blue staining (n=4). **B.** GAPD knockdown efficiency was determined at 48 hours post-nucleofection with 1μM and 1.5μM *GAPD* siRNA (n=4). Bars represent mean ± SEM.

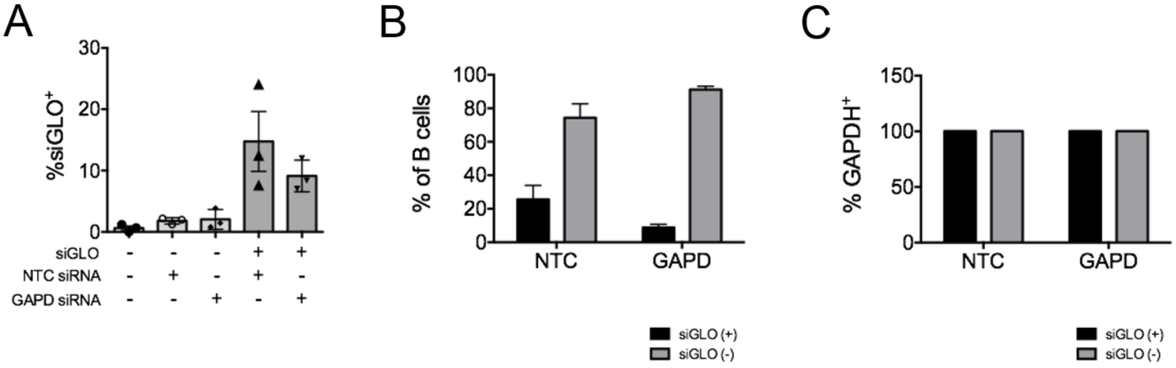

SF4. Tracking of GAPD knockdown with siGLO. Isolated human naïve B cells were co-nucleofected with siGLO and non-targeting control (NTC) siRNA or *GAPD* siRNA and rested for 24h. **A.** % siGLO^{+} B cells from **Fig. 6E** are shown at 24 hours post-nucleofection (n=3). **B.** % CD19^{+}IgD^{-} B cells from **Fig. 6F** that are siGLO^{+} and siGLO^{-} (n=3). **C.** % GAPD^{+} cells from **Fig. 6F**.
